# Invasive cardiovascular magnetic resonance (iCMR) for diagnostic right and left heart catheterization using an MR-conditional guidewire and passive visualization in congenital heart disease

**DOI:** 10.1186/s12968-020-0605-9

**Published:** 2020-03-26

**Authors:** Surendranath R. Veeram Reddy, Yousef Arar, Riad Abou Zahr, Vasu Gooty, Jennifer Hernandez, Amanda Potersnak, Phillip Douglas, Zachary Blair, Joshua S. Greer, Sébastien Roujol, Mari Nieves Velasco Forte, Gerald Greil, Alan W. Nugent, Tarique Hussain

**Affiliations:** 1 Department of Pediatrics, University of Texas Southwestern Medical Center, 5323 Harry Hines Blvd, Dallas, TX 75390 USA; 2 Pediatric Cardiology, Children’s Medical Center Dallas, 1935 Medical District Dr, Dallas, TX 75235 USA; 3 Department of Biomedical Engineering, School of Biomedical Engineering and Imaging Sciences, King’s College London, London, UK; 4 Department of Radiology, University of Texas Southwestern Medical Center, 5323 Harry Hines Blvd, Dallas, TX 75390 USA; 5 Ann & Robert H. Lurie Children’s Hospital of Chicago, 225 E Chicago Ave, Chicago, IL 60611 USA

**Keywords:** Congenital heart disease, Interventional CMR, Cardiac catheterization, Magnetic resonance imaging, Device tracking

## Abstract

**Background:**

Today’s standard of care, in the congenital heart disease (CHD) population, involves performing cardiac catheterization under x-ray fluoroscopy and cardiac magnetic resonance (CMR) imaging separately. The unique ability of CMR to provide real-time functional imaging in multiple views without ionizing radiation exposure has the potential to be a powerful tool for diagnostic and interventional procedures. Limiting fluoroscopic radiation exposure remains a challenge for pediatric interventional cardiologists.

This pilot study’s objective is to establish feasibility of right (RHC) and left heart catheterization (LHC) during invasive CMR (iCMR) procedures at our institution in the CHD population. Furthermore, we aim to improve simultaneous visualization of the catheter balloon tip, MR-conditional guidewire, and cardiac/vessel anatomy during iCMR procedures.

**Methods:**

Subjects with CHD were enrolled in a pilot study for iCMR procedures at 1.5 T with an MR-conditional guidewire. The CMR area is located adjacent to a standard catheterization laboratory. Using the interactive scanning mode for real-time control of the imaging location, a dilute gadolinium-filled balloon-tip catheter was used in combination with an MR-conditional guidewire to obtain cardiac saturations and hemodynamics. A recently developed catheter tracking technique using a real-time single-shot balanced steady-state free precession (bSSFP), flip angle (FA) 35–45°, echo time (TE) 1.3 ms, repetition time (TR) 2.7 ms, 40° partial saturation (pSAT) pre-pulse was used to visualize the gadolinium-filled balloon, MR-conditional guidewire, and cardiac structures simultaneously. MR-conditional guidewire visualization was enabled due to susceptibility artifact created by distal markers. Pre-clinical phantom testing was performed to determine the optimum imaging FA-pSAT combination.

**Results:**

The iCMR procedure was successfully performed to completion in 31/34 (91%) subjects between August 1st, 2017 to December 13th, 2018. Median age and weight were 7.7 years and 25.2 kg (range: 3 months – 33 years and 8 – 80 kg). Twenty-one subjects had single ventricle (SV) anatomy: one subject was referred for pre-Glenn evaluation, 11 were pre-Fontan evaluations and 9 post-Fontan evaluations for protein losing enteropathy (PLE) and/or cyanosis. Thirteen subjects had bi-ventricular (BiV) anatomy, 4 were referred for coarctation of the aorta (CoA) evaluations, 3 underwent vaso-reactivity testing with inhaled nitric oxide, 3 investigated RV volume dimensions, two underwent branch PA stenosis evaluation, and the remaining subject was status post heart transplant. No catheter related complications were encountered. Average time taken for first pass RHC, LHC/aortic pull back, and to cross the Fontan fenestration was 5.2, 3.0, and 6.5 min, respectively. Total success rate to obtain required data points to complete Fick principle calculations for all patients was 331/337 (98%). Subjects were transferred to the x-ray fluoroscopy lab if further intervention was required including Fontan fenestration device closure, balloon angioplasty of pulmonary arteries/conduits, CoA stenting, and/or coiling of aortopulmonary (AP) collaterals.

Starting with subject #10, an MR-conditional guidewire was used in all subsequent subjects (15 SV and 10 BiV) with a success rate of 96% (24/25). Real-time CMR-guided RHC (25/25 subjects, 100%), retrograde and prograde LHC/aortic pull back (24/25 subjects, 96%), CoA crossing (3/4 subjects, 75%) and Fontan fenestration test occlusion (2/3 subjects, 67%) were successfully performed in the majority of subjects when an MR-conditional guidewire was utilized.

**Conclusion:**

Feasibility for detailed diagnostic RHC, LHC, and Fontan fenestration test occlusion iCMR procedures in SV and BiV pediatric subjects with complex CHD is demonstrated with the aid of an MR-conditional guidewire. A novel real-time pSAT GRE sequence with optimized FA-pSAT angle has facilitated simultaneous visualization of the catheter balloon tip, MR-conditional guidewire, and cardiac/vessel anatomy during iCMR procedures.

## Background

Cardiac catheterization under x-ray fluoroscopy has been the gold standard modality for obtaining hemodynamic data in the congenital heart disease (CHD) population. The radiation burden for these subjects is becoming a more significant consideration given the increased life expectancy that we have witnessed over the past several decades. As a congenital heart center, we are now actively working towards reducing the radiation exposure and its detrimental effects which can increase carcinoma risk in the adult congenital heart disease (ACHD) population [1–7].

Congenital cardiologists are targeting increasingly complex pathologies for minimally invasive catheter-based therapies [8–10]. By improving cardiovascular magnetic resonance (CMR)-guidance, the eventual is to advance CMR-guided cardiac interventions not previously possible with x-ray fluoroscopy alone [11–15]. The unique ability of CMR to provide real-time functional imaging in multiple views without ionizing radiation exposure has the potential to be a powerful tool for diagnostic and interventional procedures.

This is a pilot study to establish feasibility of right (RHC) and left heart catheterization (LHC) during invasive CMR (iCMR) procedures at our institution in the CHD population. Our hypothesis was that successful iCMR procedures could be safely performed to determine predefined pressures and saturations. Furthermore, we aim to improve simultaneous visualization of the catheter balloon tip, MR-conditional guidewire, and cardiac/vessel anatomy during iCMR procedures.

## Methods

This is a pilot study performing real-time iCMR procedures in the Phillips Ingenia 1.5 T system (Philips Healthcare, Best, Netherlands) for RHC as well as retrograde and prograde LHC in the CHD population. We test our hypothesis that this can successfully and safely be performed by completing three tasks:

A) Perform phantom studies to identify optimal pulse sequence parameters for iCMR procedures.
B) Assess the ability of the proposed CMR sequences in a defined workflow to adequately visualize anatomy, gadolinium-filled balloon, and guidewire simultaneously.
C) Assess overall procedural success in vivo using the pulse sequence along with the proposed workflow.

### PART a: preclinical phantom testing

A novel passive catheter tracking technique using a real-time single-shot balanced steady-state free precession (bSSFP) with flip angle (FA) 35°, echo time (TE) 1.3 ms, repetition time (TR) 2.7 ms, and a non-selective partial saturation (pSAT) pre-pulse [16] was used to visualize the gadolinium-filled balloon [17], MR-conditional guidewire, and cardiac structures simultaneously. The field of view was 350 × 350 mm with a spatial resolution of 2.75 × 3.5 mm. The slice thickness ranged from 8 to 10 mm with a bandwidth of 1.5 kHz. A half scan factor of 0.625 was used to achieve a scan duration of 182 ms, and a frame rate of 5–6 frames per second. We used only passive MR-conditional catheters and guidewire. While these may have limited visibility, they are not susceptible to heating [18, 19].

**Fig. 1:**
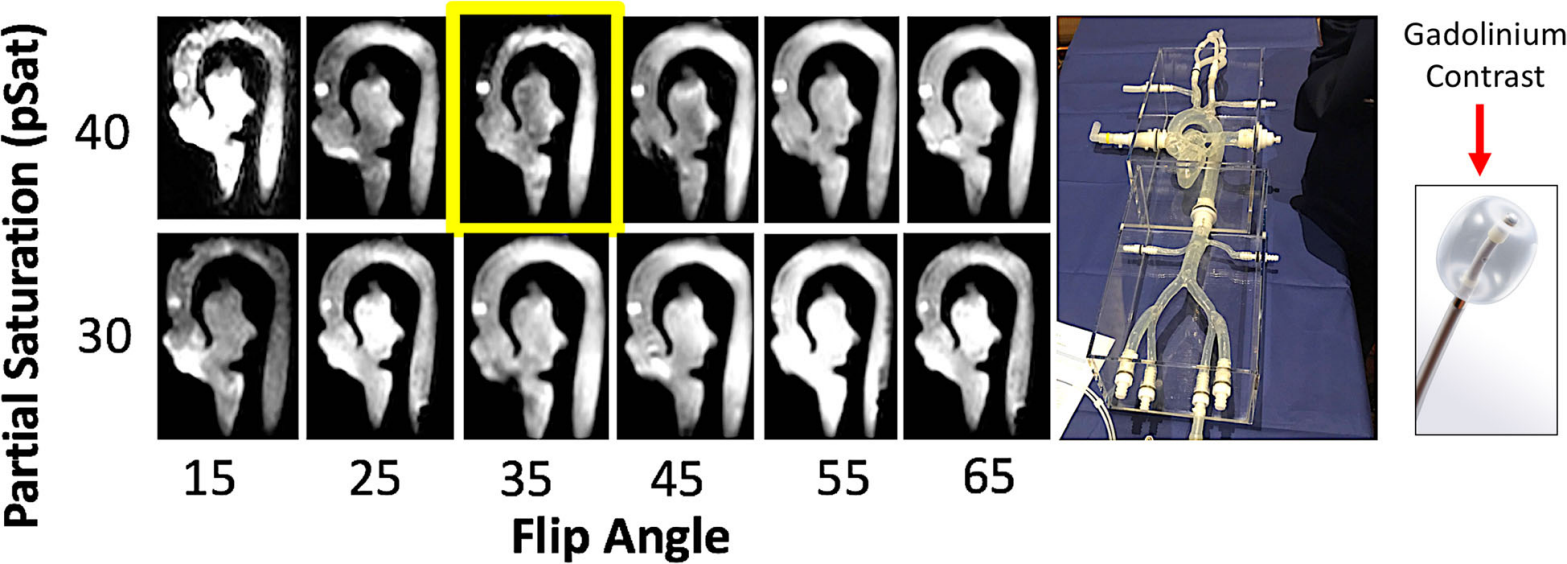
In vitro phantom testing in a cardiac model (right) filled with normal saline and a 6F gadolinium-filled balloon wedge catheter to determine the ideal flip angle (FA)-partial saturation (pSAT) angle combination for interventional cardiovascular magnetic resonance (iCMR) procedures. ***Yellow Box****– ideal* FA-pSAT angle combination *for visualization of the balloon*

Prior to human use, we performed a phantom test with a cardiac model to determine the optimal flip angles for the pSAT pulse and acquisition. The phantom was filled with normal saline and positioned within the magnet. The operator sequentially introduced the gadolinium-filled balloon followed by an MR-conditional guidewire (Emeryglide MRWire) in the phantom. Multiple images are taken at different sequence flip angles and pSAT flip angles. Images were post-processed and organized in a table for blind reviewers to qualitatively select the most optimal image settings outlined in Fig. 1.

### PART B: clinical workflow & assessment of visualization

#### Study population

The protocol was approved by the Institutional Review Board (IRB) (STU 032017–061), and was performed in the Children’s Health Medical Center CMR catheterization suite at Children’s Medical Center (CMC) in Dallas, TX. In this protocol, we conducted heart catheterization using real-time CMR in pediatric and adult research subjects already undergoing clinically-indicated heart catheterization. In many subjects, CMR was also clinically indicated (for example required for surgical or interventional planning). If CMR was not required for clinical purposes, only CMR function and flow measurements were acquired as a research procedure. After discussing the indications for and risks of the procedure, prospective informed consent and assent were obtained from all subjects and/or legal guardians as appropriate for all study related procedures.

### iCMR protocol

#### CMR environment

##### Hemodynamic recording

During the iCMR procedure, the interventionalist has direct visualization of catheter derived pressures and MR images via the bedside PRiMEGen system (National Institutes of Health (NIH), Bethesda, Maryland, USA) [20] seen in Additional file 1: Figure S1-C. This system feeds into a standard Siemens Sensis Hemodynamic recording system (Siemens Healthineers, Munich, Germany). This recording is projected in-room by using a shielded projector system [standard projector system for Philips in-room Ambient system ambiance (Philips Healthcare, Best, Netherlands)] to depict CMR images.

##### Visualization

**Fig. 2:**
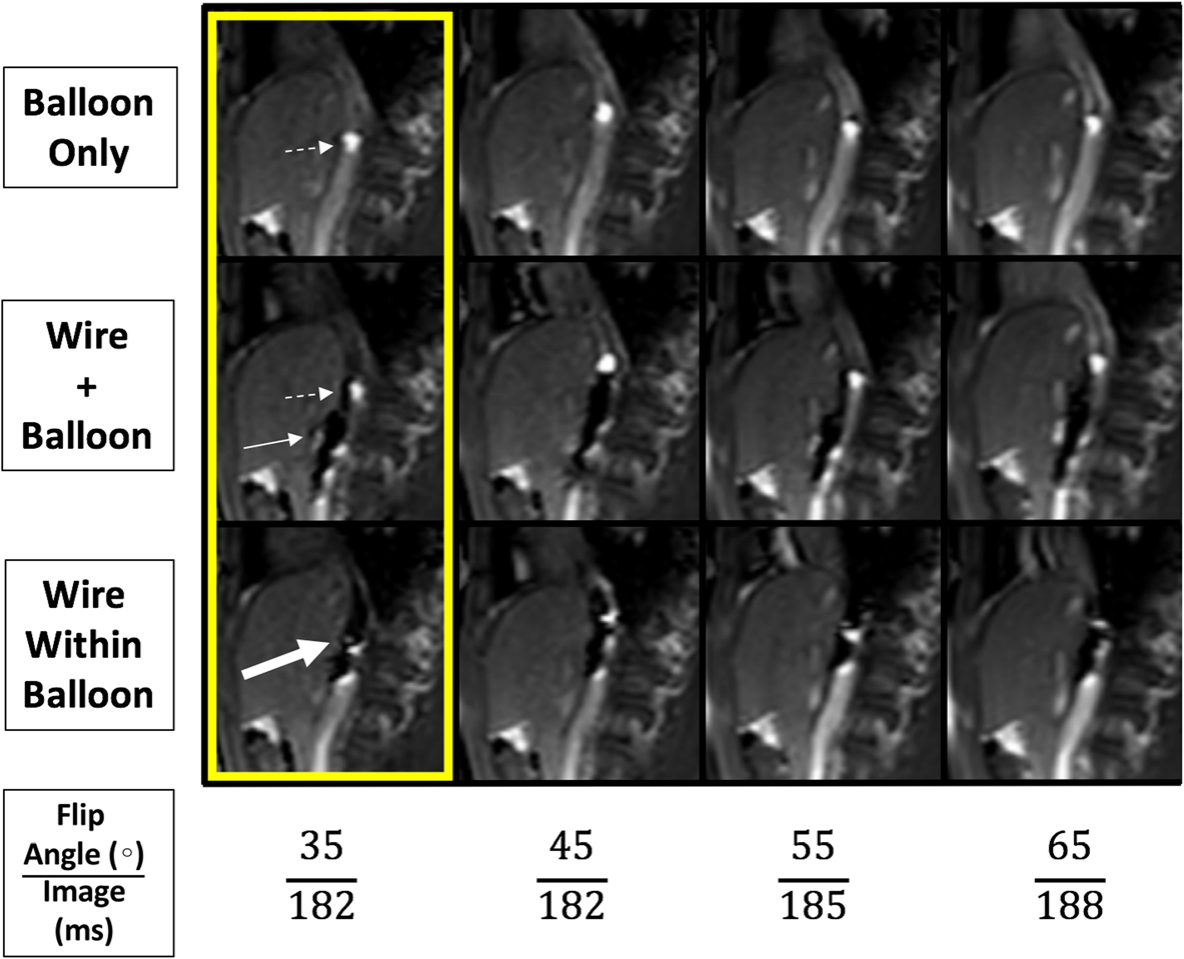
Figure depicts a novel passive tracking technique using a pSAT angle of 40^°^ at different flip angles to optimize imaging for iCMR. The upper row shows the gadolinium-filled balloon alone, the middle row shows the gadolinium-filled balloon and MR-conditional guidewire separate but within the same image, and the bottom row shows an MR-conditional guidewire markers within the gadolinium-filled balloon. The imaging acquisition scan duration time (ms) begins to increase at a FA > 45^°^. ***Dashed White Arrow****– Gadolinium-filled balloon;****Solid White Arrow****– MR-conditional guidewire;****Thick White Arrow****– MR-conditional guidewire markers within the gadolinium-filled balloon.****Yellow box****- Ideal* in-vivo *flip angle combination* (pSAT angle = 40^°^; FA = 35^°^) to simultaneously visualize the catheter balloon tip, MR-conditional guidewire, and cardiac/vessel anatomy during iCMR procedures. *Note*: No decrease in image acquisition time was seen at FA < 45^°^

Using the Philips interactive scanning mode (Philips Healthcare), the CMR technologist can modify the imaging slice location throughout the scan to maintain visualization of the catheter as it is advanced by the bedside operator. Using the optimized flip angles for the pSAT pulse and the acquisition from our preclinical testing (cardiac model shown in Fig. 1), we trialed in vivo CMR-guided catheterization with results outlined in Fig. 2.

Prior to introducing catheters, the imaging team planned and stored geometries (‘image stamps’) that will be required for real-time catheter visualization and manipulation. For example, this may include an inferior vena cava stamp, sagittal bicaval stamp, right ventricle inflow-outflow stamp, axial branch pulmonary artery stamp, para-sagittal aortic arch stamp and/or coronal left ventricular outflow stamp.

##### MR-conditional catheter and guidewire

**Fig. 3:**
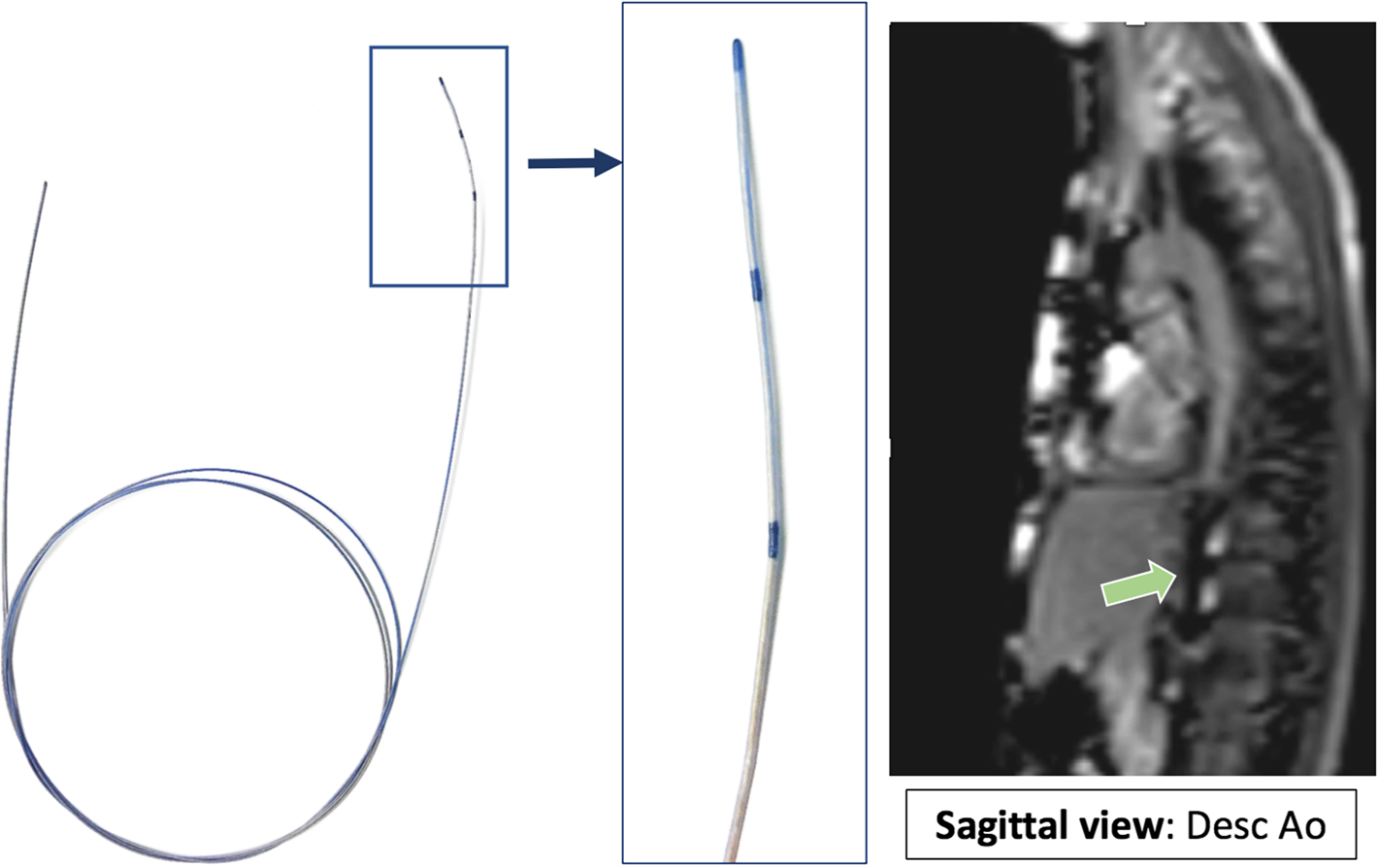
FDA cleared and CE marked MR conditional wire (0.035″ diameter) with three nanoparticle markers at discrete positions located at the tip, 2 and 4 cm from the end producing significant passive susceptibility artifact. ***Green Arrow*** – MR-conditional guidewire artifact in a sagittal view midway up the descending aorta aiding in a retrograde LHC

A 6-French Arrow, Balloon Wedge-Pressure Catheter (Teleflex Medical Headquarter International, Ireland, Model #: AI-07124 and/or AI-07126) was used for all procedures. The balloon-tip of the catheter was filled with dilute gadolinium (1-part gadolinium to 99-parts saline) and guided with the help of an MR-conditional guidewire to specific structures in the heart to obtain necessary hemodynamics. This guidewire is United States Food and Drug Administration (FDA) cleared and Conformité Européenne (CE) marked MR-conditional guidewire (angled-tip Emeryglide MRWire, Nano4Imaging, Aachen, Germany). The angled-tip MR-conditional guidewire used is 0.035″ (0.89 mm) in diameter and 180 cm in length (Fig. 3). At the soft end of the guidewire, there are three passive markers caused by the nanoparticle coating that produces a distinct susceptibility artifact (0 mm, 20 mm, and 40 mm from the tip). These markers allow for passive visualization during real-time CMR. The wire is made of a fiber reinforced composite with double internal winding and the entire body/tip are packed into one sleeve to prevent detachment of tip and breaking of the internal core. This MR-conditional guidewire was made available to our institution starting with subject #10 **(**Additional file 2: Table S1).

### PART C: procedural success and safety

iCMR procedural success was defined as a full study where all predefined pressure and saturation goals were met. In general, an iCMR procedure was considered to be successfully completed if all catheter derived hemodynamics (pressures and saturations to calculate flows and resistances) and CMR derived flow and volume data (with anatomic/morphologic information when indicated) was obtained. An MR-conditional guidewire utilization was deemed as successful if the wire was used successfully to obtain catheter access to predetermined locations. Additional variables monitored were subjective quality of an MR-conditional guidewire and gadolinium-filled balloon visualization in the magnet by operators, time for first pass RHC and LHC, as well as total iCMR anesthesia time. Safety outcomes were further broken down into anesthesia, catheter, or CMR-related. All safety measures and follow up criteria were in accordance with IMPACT (IMproving Pediatric and Adult Congenital Treatment) registry data collection standards.

## Results

### PART a: preclinical phantom testing

Qualitative observational review was performed with three operators to determine the optimal FA-pSAT angle combination to visualize the gadolinium-filled balloon within the in vitro cardiac phantom model (Fig. 1). Pre-clinical imaging correlated with subsequent in vivo testing. The ideal in vivo flip angle combination was again found to be a pSAT angle of 40°, and a FA of 35°.

### PART B: clinical workflow & assessment of visualization

#### Subject demographics and indications

The iCMR procedure was performed in 34 subjects at our institution (Table 1). Twenty-one subjects had single ventricle (SV) anatomy: one subject referred for pre-Glenn evaluation, 11 pre-Fontan evaluations and 9 post-Fontan subjects for protein losing enteropathy (PLE) and/or cyanosis evaluations. Thirteen subjects had bi-ventricular (BiV) anatomy: 4 referred for coarctation of the aorta (CoA) evaluations, 3 underwent vaso-reactivity testing with inhaled nitric oxide, 3 investigated for right ventricular (RV) volumes, two underwent branch pulmonary artery (PA) stenosis evaluation, and the remaining subject was status post heart transplant.

**Table 1:** Characteristics for all iCMR subjects

**MRI-Conditional Guidewire Patients (*n*** **= 34)**	
**Demographic data**	
Sex (%) - 74% male (*n* = 25)	
Age - 7.7 years (range: 3 months - 33 years)	
Weight (kg) - 25.2 (range: 8–80)	
Single Ventricle (%) - 62%	
**Single Ventricle, n = 21**	
Post-Fontan = (9/21) = 43%	
Pre-Fontan = (11/21) = 52%	
Pre-Glenn = (1/21) = 5%	
**Biventricular,*n*** **= 13**	
Coarctation of the Aorta = (4/13) = 31%	
Vasoreactivity testing = (3/13) = 23%	
Tetralogy of Fallot = (3/13) = 23%	
Pulmonary Artery Stenosis = (2/13) = 15%	
s/p Heart Transplant = (1/13) = 8%	
**Procedure Time**	
**Single Ventricle,*n*** **= 21**	
Total Zone 4 time = 129 min (59–174)	
Right heart cath = 5.0 min	
Left heart cath = 2.9 min	
**Biventricular, n = 13**	
Total Zone 4 time = 124 min (57–259)	
Right heart cath = 5.4 min	
Left heart cath = 3.1 min	
**Outcomes**	
**Single Ventricle, n = 21**	
Successful, *n* = 19	
Unsuccessful, *n* = 1 due to poor visualization	
Unsuccessful, *n* = 1 due to coil artifact	
**Biventricular, n = 13**	
Successful, *n* = 12	
Unsuccessful, n = 1 due to inability to cross CoA	

### Visualization

In vivo testing (Fig. 2) confirmed the sequence parameters chosen during pre-clinical testing (pSAT 40°/ FA 35°). Using the given sequence and workflow the Gadolinium-filled balloon visualization was adequate for all cases (except one case where the partial saturation pulse was inadvertently disabled and one case with significant artifact from implanted devices: these are outlined below). Additional file 3: Figure S3 outlines the total percentage of gadolinium-filled balloon visualization during CMR-guided real-time catheterization using the interactive scanning mode. On average, the gadolinium-filled balloon is seen 45.3% of the time with a standard deviation of 10.6 through our first 25 cases with an MR-conditional guidewire. The percentage of time does not significantly improve with subsequent cases. Furthermore, two cases had suboptimal visualization (described below). An MR-conditional guidewire produced a significant susceptibility artifact and was identified in all cases using the described sequences and workflow. In the x-ray fluoroscopy lab, an MR-conditional guidewire shaft and three distal markers are not readily conspicuous (Additional file 5: Figure S4).

### PART C: procedural success and safety

#### Overall success

**Fig. 4:**
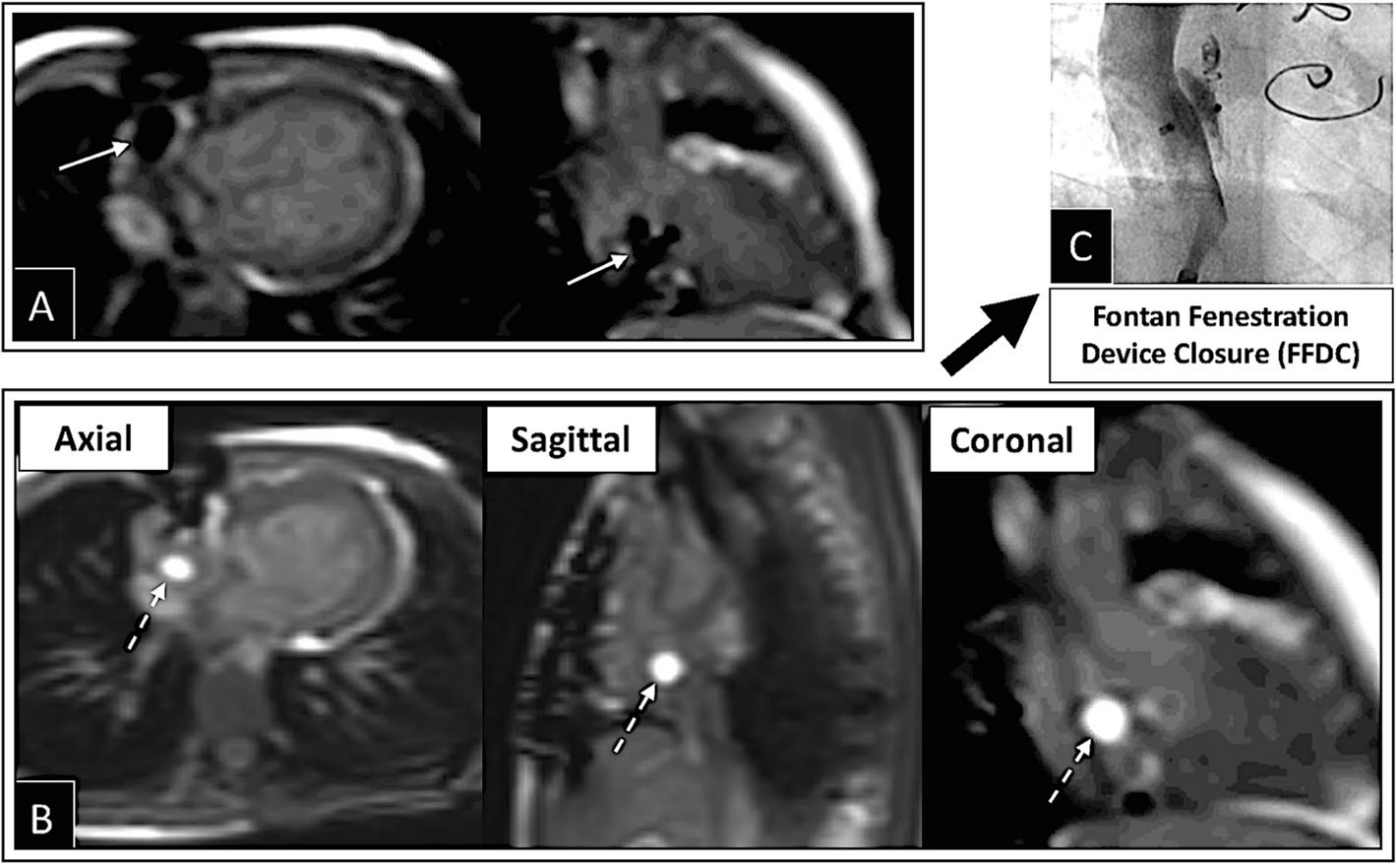
Depicts successful iCMR Fontan fenestration test occlusion (FFTO) followed by successful Fontan fenestration device closure (FFDC) in the x-ray fluoroscopy cath lab. **a** Under iCMR, the Fontan fenestration (FF) was crossed using an MR-conditional guidewire. An MR-conditional guidewire is seen across the Fontan fenestration and in the RA appendage. **b** The gadolinium-filled balloon was deflated to advance across the FF, inflated again in the right/pulmonary venous atrium and then pulled back to perform FFTO. Cath and CMR hemodynamics were repeated with FFTO. **c** If deemed appropriate, the subject was transferred to the x-ray fluoroscopy lab for FFDC. ***Solid White Arrow****– MR-conditional guidewire;****Dashed White Arrow****– Gadolinium-filled balloon*

The iCMR procedure was successfully performed to completion in 31/34 (91%) subjects between August 1st, 2017 to December 13th, 2018. Time taken for first pass RHC, LHC/aortic pull back, and to cross/confirm the catheter across the Fontan fenestration was 5.2, 3.0, and 6.5 min, respectively. Total success rate to obtain required data points to complete Fick principle calculations for all patients was 331/337 (98%). Transfer to the x-ray fluoroscopy lab for intervention was required in 24/34 iCMR subjects. Interventions included Fontan fenestration device closure (Fig. 4), balloon angioplasty of conduits/pulmonary arteries, CoA stenting, and/or coiling of collaterals when indicated.

#### MR-conditional guidewire catheterization outcomes

**Fig. 5:**
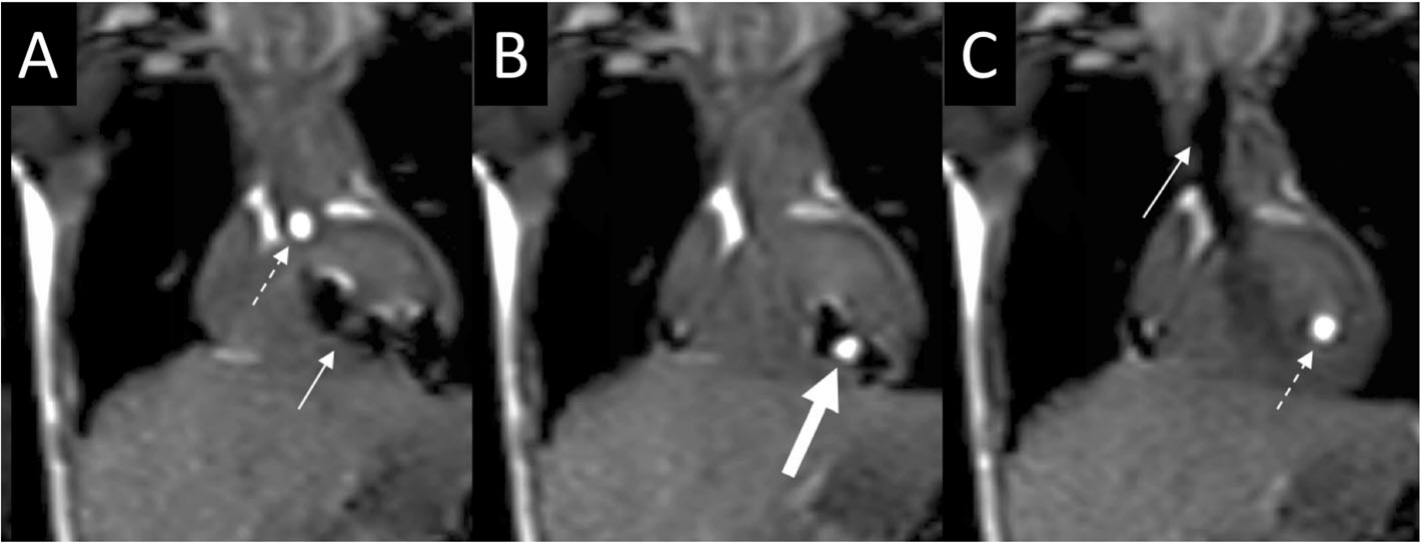
Coronal interactive series depicting retrograde entry into the LV during a LHC. **a** The gadolinium-filled balloon (*dashed white arrow*) is seen at the aortic root as an MR-conditional guidewire (*solid white arrow*) is seen in the LV. **b** An MR-conditional guidewire is within the gadolinium-filled balloon (*thick white arrow*) in the LV. **c** The gadolinium-filled balloon remains in the LV to measure pressures as an MR-conditional guidewire is withdrawn to the ascending aorta; ***Dashed White Arrow****– Gadolinium-filled balloon;****Solid White Arrow****– MR-conditional guidewire;****Thick White Arrow****– MR-conditional guidewire within the gadolinium-filled balloon*

**Fig. 6:**
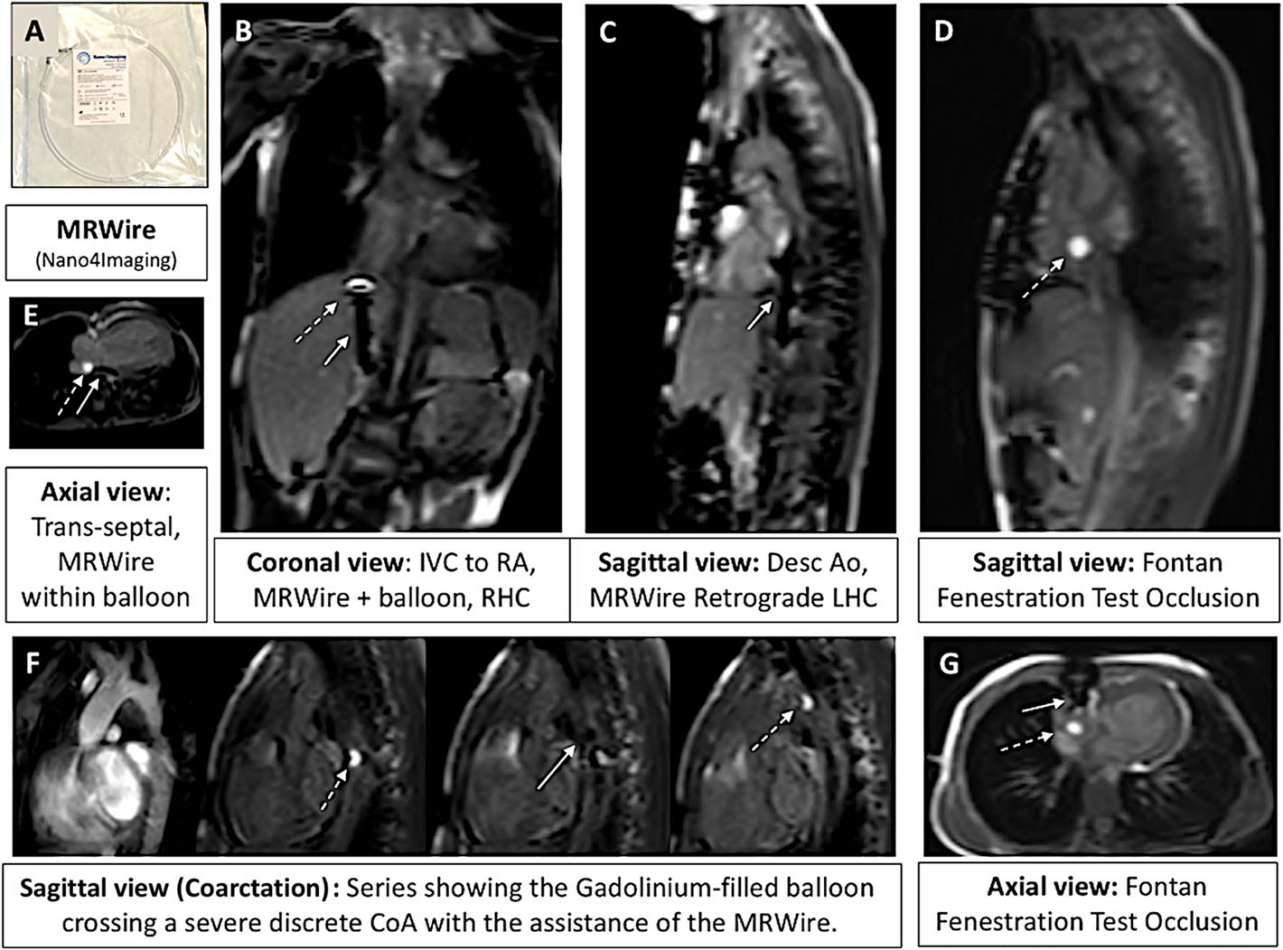
**a** MR-conditional guidewire (angled-tip Emeryglide MRWire, Nano4Imaging, Aachen, Germany). **b** Coronal view of an MR-conditional guidewire (*solid white arrow*) being used to guide the gadolinium-filled balloon (*dashed white arrow*) for a RHC. **c** MR-conditional guidewire used for a retrograde LHC. **d** Gadolinium-filled balloon used for successful Fontan Fenestration Test Occlusion (FFTO). **e** Depiction of an MR-conditional guidewire being used to guide the gadolinium-filled balloon across a pre-existing atrial communication in order to enter the LA obtaining pulmonary venous saturations. **f** Series showing the gadolinium-filled balloon crossing a severe discrete CoA with the assistance of an MR-conditional guidewire. **g** Axial view of the previously depicted FFTO. ***Dashed White Arrow****– Gadolinium-filled balloon;****Solid White Arrow****– MR-conditional guidewire*

Starting with subject #10, an MR-conditional guidewire was used in all subsequent subjects with a success rate of 96% (24/25). We were able to successfully perform RHC, trans-septal (pre-existing atrial communication) assisted prograde LHC, retrograde LHC/aortic pullback (Fig. 5), obtain pulmonary venous saturations, cross stenotic areas (stenotic RV-PA conduit, stenotic branch pulmonary arteries and CoA sites), and perform Fontan fenestration test occlusion under real-time CMR visualization (Fig. 6). Real-time CMR-guided RHC (25/25 subjects, 100%), retrograde and prograde LHC/aortic pull back (24/25 subjects, 96%), CoA crossing (3/4 subjects, 75%) and Fontan fenestration test occlusion (2/3 subjects, 67%) were successfully performed in the majority of subjects when an MR-conditional guidewire was utilized. The unsuccessful CoA case was the only unsuccessful LHC in this cohort. Except for the arrhythmia described in subject #34 (below), no other complications were encountered.

When using the MR-conditional guidewire, there was only one iCMR case that was deemed to be unsuccessful in gaining all predefined pressures and saturations in our cohort of 25 subjects. This case was related to an MR-conditional guidewire, as the wire was unable to aid in advancing the catheter across a severe CoA site to obtain ascending aortic pressures and coarctation gradient. One case did not have advanced testing performed with fenestration occlusion due to arrhythmia. All subjects were admitted for overnight observation and monitored as an outpatient until their next follow up clinic visit with their primary cardiologist. Study coordinators reached out to families within a week of the procedure to ensure there were no complications or further concerns.

#### Unsuccessful related to suboptimal visualization

##### Disabled partial saturation (pSAT)

*Subject #8* case was considered successful in getting predetermined saturations and pressures. However, there was poor visualization due to unexpected loss of pSAT pulse during the procedure. This resulted in difficulty visualizing the gadolinium-filled balloon at times. This was a software issue pertaining to the previous CMR platform version and has been subsequently rectified by the vendor. No further cases have been affected due to the gadolinium-filled balloon’s visualization since that time.

##### CMR visualization (coil artifact)

*Subject #30* case was considered successful in getting predetermined saturations and pressures. However, there was poor visualization secondary to significant coil artifact. The subject was referred from an outside institution with multiple stainless steel coils (Cook Cardiology, Bloomington, Indiana, USA) placed in bilateral internal mammary arteries. Unfortunately, the artifact produced by these coils led to significant difficulty to accurately measure the CMR flows and the iCMR procedure was aborted as soon as the catheter-based hemodynamics were obtained to calculate Fick principle measurements.

#### Unsuccessful related to MR-conditional guidewire

##### MR-conditional guidewire size

*Subject #27’s* case was unsuccessful due to inability of an MR-conditional guidewire to pass a tortuous CoA. We deemed this case as unsuccessful and related to an MR-conditional guidewire, even though all flows and resistances were calculated except for pressure gradient across the severe CoA. Multiple attempts were made to advance the 0.035″ MR-conditional guidewire across the area of severe CoA, and at one point the wire seemed to have made its way past the CoA into the left subclavian artery but the catheter could not be advanced across the CoA site to the ascending aorta. In addition, an MR-conditional guidewire passage across the CoA could not be confirmed by CMR imaging due to the limited spatial resolution of the real-time imaging sequence. Further attempts were aborted. After transfer to the x-ray fluoroscopy lab, the CoA site was crossed with a soft 0.018″ wire. In the future, subjects with this anatomy would benefit from a smaller diameter CMR-conditional guidewire. Performing CMR angiography in these cases during preparation prior to catheterization may be valuable.

##### MR-conditional guidewire-induced arrhythmia during advanced testing

S*ubject #34’s* case was considered successful in getting predetermined saturations and pressures. However, advanced testing (Fontan fenestration test occlusion procedure) could not be completed in the CMR suite. The iCMR procedure was aborted due to transient hypotension secondary to wire-provoked intra-atrial reentrant tachycardia (IART) after crossing the Fontan fenestration with an MR-conditional guidewire. The subject was known to suffer from this arrhythmia previously and was on Sotalol for prevention. IART was noted as soon as an MR-conditional guidewire was advanced across the Fontan fenestration. The subject was immediately moved from Zone 4 to Zone 3 in the CMR suite and underwent successful synchronized cardioversion on the first attempt with 20 J. The time from arrhythmia initiation to successful cardioversion was less than 90 s. There were no clinical sequelae related to the event. The subject was transferred to the x-ray fluoroscopy lab where a repeat Fontan fenestration test occlusion was performed with no complications. As the subject was relatively asymptomatic, the Fontan fenestration was not closed to preserve the communication for a potential EP study/ablation of the IART pathways.

##### Anesthesia timing

Total anesthesia time was closely monitored from start to finish of the all iCMR procedures. Overall, anesthesia time decreased by approximately 15 min per case over the first 10 cases (Additional file 4: Figure S2). This can be attributed to improved workflow, staff comfort with the procedure, and streamlined roles. Average total anesthesia time was 125.32 min (range 59 to 297 min) from subject entering and leaving Zone 4 of the CMR suite where the iCMR procedure is undertaken.

##### Safety considerations

Safety outcomes were monitored during and shortly after the iCMR procedure. The CMR table top was unintentionally moved in subjects #5 and #6 due to the operator leaning on the control panel when advancing the wedge catheter in smaller subjects (Additional file 1: Figure S1-D). No complications were encountered during or shortly after the bed movement. Due to these events, a plastic covering was constructed and placed over the control panel. Since that time, we have had no further unintentional table top movement during the procedure. No other anesthesia related, CMR-related and/or catheter-based complications have been encountered including no inadvertent extubation, catheter dislodgement, hematoma, magnet-induced increase in body temperature, or pressure sores/ulcers during the patient encounter.

## Discussion

Our data demonstrate feasibility for iCMR-directed RHC, LHC, pulmonary venous saturation assessment, CoA diagnostics, and Fontan fenestration test occlusion hemodynamic testing. We found an MR-conditional guidewire especially helpful to direct the wedge catheter into small vessels. By adding the combined use of novel imaging sequences with an MR-conditional guidewire, we can increase the success rate for complete radiation-free diagnostics when compared to previous studies [21] with a 96% success rate in our subject cohort that is predominantly comprised of complex CHD subjects.

From our experience, the success of iCMR procedures is heavily dependent upon reliable visualization of the dilute gadolinium-filled balloon-tip catheter and MR-conditional guidewire. The recently developed catheter tracking technique using a real-time bSSFP with pSAT pre-pulse [16] helped improve balloon visualization by increasing the contrast between the gadolinium and surrounding tissue. While bSSFP sequences generally use a higher FA (50–80°) to maximize signal from the blood, a lower FA (35°-45°) was found to be a good tradeoff between signal-to-noise ratio (SNR) and further selective reduction of the blood pool signal. Since the signal generated by the bSSFP sequence peaks at different FA for different tissues [22], reducing the FA to this lower range has the effect of simultaneously reducing fluid signal (and other tissues with larger (T2/T1) ratios), while maintaining signal from tissues with smaller (T2/T1) ratios, such as the liver and muscle (Fig. 2). Pre-clinical phantom testing made optimization of these sequence parameters possible, allowing for concurrent visualization of the bright gadolinium-filled balloon in the vasculature, the surrounding anatomy, and the susceptibility artifacts caused by the guidewire markers. This created a more reliable iCMR environment and enabled our team to successfully monitor and guide instruments for complete cardiac evaluations.

Previously reported passive catheter tracking strategies include carbon dioxide filled balloons [23, 24], positive contrast balloons [25], saturation on/off pulses [26], and dual echo bSSFP for positive balloon and guidewire contrast [26]. The demonstrated pSAT catheter imaging sequence has the added benefit of providing positive balloon contrast with simultaneous visualization of the underlying anatomy, without the need to disable the pre-pulse. Active tracking approaches [27, 28] can significantly improve device visualization, although such devices have been largely restricted to preclinical studies and have been successfully used for EP applications [29–31].

Another consideration is the rate of image acquisition during fluoroscopy in the CMR suite versus x-ray lab. The imaging acquisition scan duration time was shown to increase at a FA > 45^°^ (Fig. 2). Our standard CMR fluoroscopy time was 5.5 frames per second (182 ms per image acquisition) which is lower than our standard x-ray fluoroscopy time of 15 frames per second (66 ms per image acquisition). This may lead to a slight delay in recognition of a complication but is compensated by increased soft tissue clarity. In addition, the authors acknowledge that x-ray fluoroscopy labs are moving toward lower frame rates in an effort to reduce overall radiation.

Advances in low specific absorption rate (SAR) imaging to allow for commercially available metallic guidewires to be used in the iCMR environment with negligible heating show promise for future applications [32]. Other CE marked MR-conditional guidewires such as the EPflex (GmbH, Dettingen, Germany) have been successfully used in Europe.

## Future directions

We will continue to explore the benefits of an MR-conditional guidewire with the goal of working toward implementing real-time quantitative assessment of interventional outcomes such as balloon angioplasties within the CMR magnet. We also continue to work towards decreasing our iCMR anesthesia time as outlined in this discussion.

Furthermore, iCMR is advantageous for the aging single ventricle population [33–35]. We are able to generate data from combined catheter-based Fick principle and CMR-derived flow hemodynamics that holds the promise to better define single ventricle physiology. Characterization of Fontan hemodynamics with and without a fenestration will lead to future prospective studies to better assess the failing Fontan circuit. We plan to conduct a prospective study to better assess suitability for fenestration closure by measuring catheter obtained Fontan pressures and systemic saturation along with accurate measurement of MR-derived cardiac output. The ultimate goal would be to collaborate with industry to develop an appropriate occlusion device to be placed in the CMR environment. In addition, iCMR studies will provide the pediatric and adult CHD practitioners a better understanding of abnormal lymphatics with dynamic contrast MR lymphangiography [36], Fontan-Associated Liver Disease through hepatic wedges and elastography, assessment of the aortopulmonary/veno-venous collateral burden, and a more accurate assessment of ventricular volumes. Newer sequences are being undertaken in the iCMR environment to improve the seamless visualization of the catheter motion and reduce the catheter/wire or coil associated artifacts.

The interactive scanning mode used during this study was found to have limitations in reliable continuous visualization of the gadolinium-filled balloon (Additional file 3: Figure S3) [16]*.* Further investigation with dedicated CMR-guided intervention systems, such as the Philips iSuite system (Philips Research, Hamburg, Germany), will allow us to view multiple projections at once and ultimately improve visualization of our gadolinium-filled balloon guided catheter and MR-conditional guidewire during iCMR procedures.

## Conclusion

Feasibility for diagnostic RHC, LHC, crossing a CoA, pulmonary vein access, and Fontan fenestration test occlusion iCMR procedures with an MR-conditional guidewire in CHD subjects is demonstrated. Novel real-time bSSFP with optimized FA-pSAT angles has facilitated simultaneous visualization of the catheter balloon tip, MR-conditional guidewire, and cardiac/vessel anatomy during iCMR procedures. The Fontan fenestration test occlusion cases also describe a more thorough evaluation of Fontan pressures and cardiac output before Fontan fenestration device closure by using accurate flow, ventricular volumes, and cardiac output measurements from real-time CMR with simultaneous catheter-based pressure measurements.

## Supplementary information

**Additional file 1: Figure S1.** A) Philips MR table top used to transfer subjects from Zone 3 to Zone 4. B) Wireless optical CMR- communication system powered by Optoacoustics C) Phillips Ingenia 1.5 Tesla Magnet with a Sensavue screen and in vivo pressure system D) Plastic covering over CMR control panel to prevent bedside operator from unintentionally moving table top during iCMR procedure.

**Additional file 2: Table S1.** iCMR subject description including age, sex, diagnosis, clinical indication, and success outcome. Starting with subject #10, the MR-conditional guidewire was used for all subsequent cases. Abbreviations: LPA left pulmonary artery, PA pulmonary artery, DKS Damus–Kaye–Stansel, AVC Atrioventricular Canal, BTS Blalock-Taussig Shunt, TOF Tetralogy of Fallot, HLHS hypoplastic left heart syndrome, RV right ventricle, PV pulmonary valve, CoA, coarctation of the aorta, T21 trisomy 21, PDA patent ductus arteriosus, iNO inhaled nitric oxide, dTGA dextro-transposition of the great arteries, HRHS hypoplastic right heart syndrome, ASD atrial septal defect, VSD ventricular septal defect, DILV double inlet left ventricle, PAB pulmonary artery banding, PLE protein losing enteropathy, IVS intact ventricular septum, OHT orthotopic heart transplant

**Additional file 3: Figure S3.** Depiction of iCMR gadolinium balloon visualization during interactive MR-guided catheterization in MR-conditional guidewire subject cases (overall subject #10–34).

**Additional file 4: Figure S2.** Depiction of decreasing anesthesia time for MR-guided catheterization. The anesthesia time decreases dramatically within the first 10 cases. We noticed an average decrease of approximately 15 min per case.

**Additional file 5: Figure S4.** Series of AP x-ray fluoroscopy images showing the extremely limited visualization of the MR-conditional guidewire in a subject with a severe discrete CoA (CoA MR images shown in Fig. 5f). A) Depicts the lack of visualization of the MR-conditional guidewire in the proximal descending aorta under x-ray fluoroscopy. B) Pigtail catheter successfully advanced over MR-conditional guidewire. *Black Arrow –* Limited visualization of MR-conditional guidewire under x-ray fluoroscopy.

**Additional file 6.** Supplemental information.

## Data Availability

The datasets used and/or analyzed during the current study are available from the corresponding author on reasonable request.

## Abbreviations

ACHD: Adult congenital heart disease
ACT: Activated clotting time
AP: aortopulmonary
BiV: Bi-ventricular
bSSFP: Balanced steady-state free precession
CE: Conformité Européenne
CHD: Congenital heart disease
CMC: Children’s Medical Center
CoA: Coarctation of the aorta
FA: Flip angle
FDA: Food and Drug Administration
IART: Intra-atrial reentrant tachycardia
iCMR: Invasive cardiovascular magnetic resonance
IMPACT: IMproving Pediatric and Adult Congenital Treatment
iNO: Inhaled nitric oxide
IRB: Institutional Review Board
LHC: Left heart catheterization
MR: Magnetic resonance
MRI: Magnetic resonance imaging
NIH/NHLBI: National Institutes of Health/National Heart, Lung, and Blood Institute
PLE: Protein losing enteropathy
pSAT: Partial saturation
RHC: Right heart catheterization
SAR: Specific absorption rate
SNR: Signal-to-noise ratio
SV: Single ventricle
TE: Echo time
TR: Repetition time
