# Supplementary figures and images for: Invasive cardiovascular magnetic resonance (iCMR) for diagnostic right and left heart catheterization using an MR-conditional guidewire and passive visualization in congenital heart disease
Source: J Cardiovasc Magn Reson. 2020 Mar 26;22:20. doi: 10.1186/s12968-020-0605-9 (PMC7098096; doi:10.1186/s12968-020-0605-9)

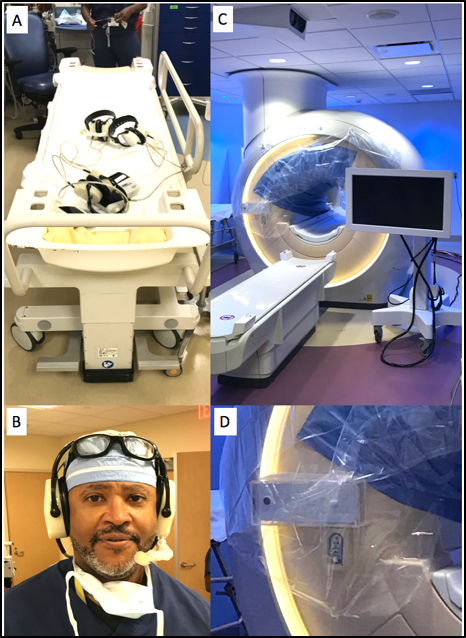

Supplement: Supplementary file 1 — Additional file 1: Figure S1. A) Philips MR table top used to transfer subjects from Zone 3 to Zone 4. B) Wireless optical CMR- communication system powered by Optoacoustics C) Phillips Ingenia 1.5 Tesla Magnet with a Sensavue screen and in vivo pressure system D) Plastic covering over CMR control panel to prevent bedside operator from unintentionally moving table top during iCMR procedure. [file 12968_2020_605_MOESM1_ESM.png]

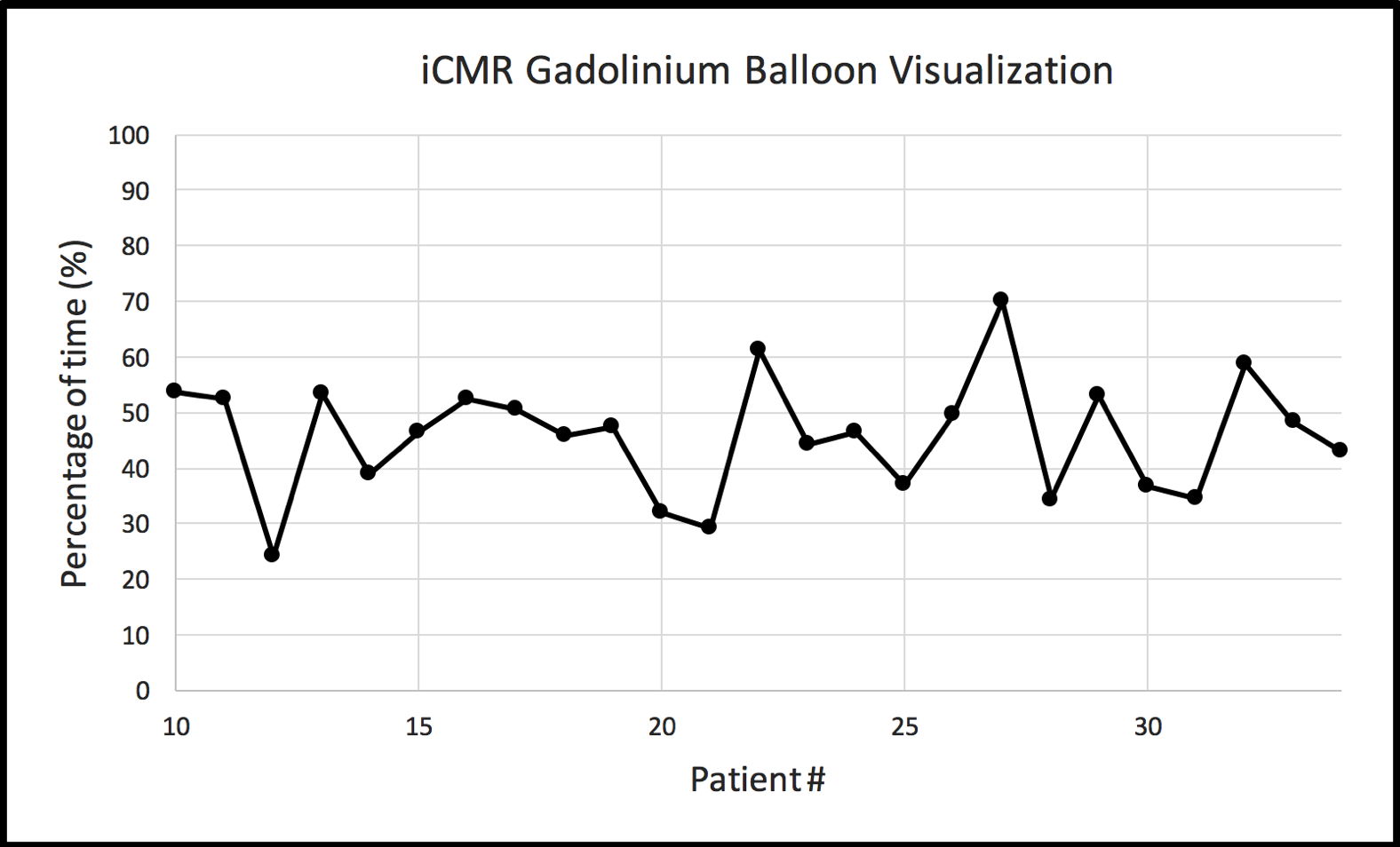

Supplement: Supplementary file 3 — Additional file 3: Figure S3. Depiction of iCMR gadolinium balloon visualization during interactive MR-guided catheterization in MR-conditional guidewire subject cases (overall subject #10–34). [file 12968_2020_605_MOESM3_ESM.png]

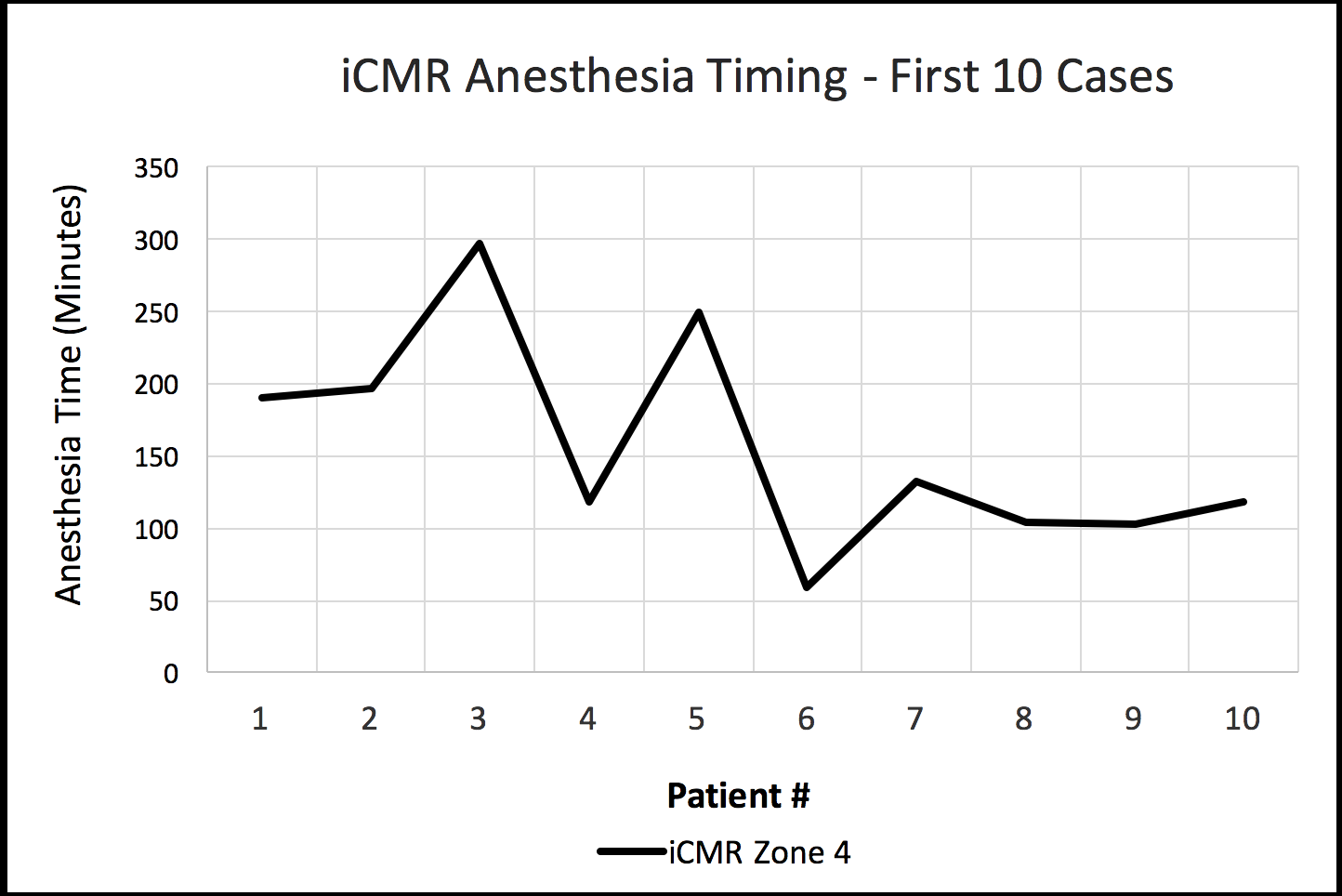

Supplement: Supplementary file 4 — Additional file 4: Figure S2. Depiction of decreasing anesthesia time for MR-guided catheterization. The anesthesia time decreases dramatically within the first 10 cases. We noticed an average decrease of approximately 15 min per case. [file 12968_2020_605_MOESM4_ESM.png]

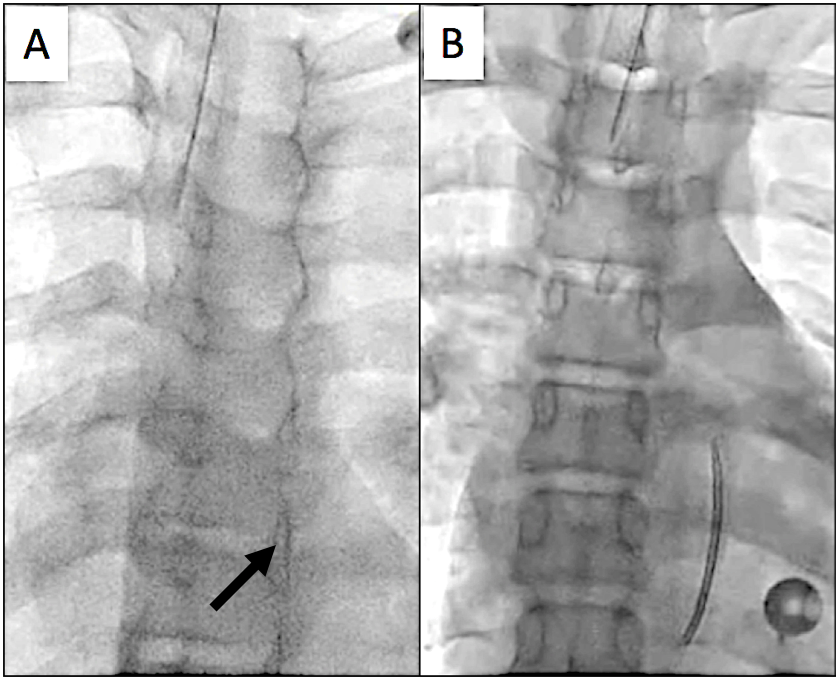

Supplement: Supplementary file 5 — Additional file 5: Figure S4. Series of AP x-ray fluoroscopy images showing the extremely limited visualization of the MR-conditional guidewire in a subject with a severe discrete CoA (CoA MR images shown in Fig. 5f). A) Depicts the lack of visualization of the MR-conditional guidewire in the proximal descending aorta under x-ray fluoroscopy. B) Pigtail catheter successfully advanced over MR-conditional guidewire. Black Arrow – Limited visualization of MR-conditional guidewire under x-ray fluoroscopy. [file 12968_2020_605_MOESM5_ESM.png]
