# Supplementary material for: Invasive cardiovascular magnetic resonance (iCMR) for diagnostic right and left heart catheterization using an MR-conditional guidewire and passive visualization in congenital heart disease
Source: J Cardiovasc Magn Reson. 2020 Mar 26;22:20. doi: 10.1186/s12968-020-0605-9 (PMC7098096; doi:10.1186/s12968-020-0605-9)
Supplement: Supplementary file 2 — Additional file 2: Table S1. iCMR subject description including age, sex, diagnosis, clinical indication, and success outcome. Starting with subject #10, the MR-conditional guidewire was used for all subsequent cases. Abbreviations: LPA left pulmonary artery, PA pulmonary artery, DKS Damus–Kaye–Stansel, AVC Atrioventricular Canal, BTS Blalock-Taussig Shunt, TOF Tetralogy of Fallot, HLHS hypoplastic left heart syndrome, RV right ventricle, PV pulmonary valve, CoA, coarctation of the aorta, T21 trisomy 21, PDA patent ductus arteriosus, iNO inhaled nitric oxide, dTGA dextro-transposition of the great arteries, HRHS hypoplastic right heart syndrome, ASD atrial septal defect, VSD ventricular septal defect, DILV double inlet left ventricle, PAB pulmonary artery banding, PLE protein losing enteropathy, IVS intact ventricular septum, OHT orthotopic heart transplant [file 12968_2020_605_MOESM2_ESM.pdf]

Patient	Age (years)	Sex	Diagnosis	Clinical Question	Success
#1	13	M	LPA stenosis	PA stenosis	Yes
#2	2	F	Anatomically corrected malposition of the great arteries s/p modified DKS s/p Glenn	Pre-Fontan	Yes
#3	0.25	M	AVC, pulmonary atresia with discontinuous PAs s/p unifocalization with BTS	1V vs 2V repair	Yes
#4	17	F	TOF s/p surgical bioprosthetic pulm valve implantation s/p LPA stent	RV volumes	Yes
#5	2	M	TOF with absent PV s/p RV-PA conduit with a 12 mm Aortic homograft with LeCompte	RV volumes	Yes
#6	5	M	HRHS + d-TGA s/p Norwood/Sano s/p Glenn s/p extracardiac fenestrated Fontan	Fenestrated Fontan	Yes
#7	7	M	HLHS s/p Glenn s/p fenestrated Fontan	Fenestrated Fontan	Yes
#8	4	M	HLHS s/p Norwood/Sano s/p Glenn	Pre-Fontan	No
#9	4	M	HLHS s/p Norwood/Sano s/p Glenn	Pre-Fontan	Yes
#10	4	M	HLHS s/p Norwood/Sano s/p Glenn	Pre-Fontan	Yes
#11	5	M	Severe Discrete CoA	Coarctation	Yes
#12	4	M	HLHS s/p Norwood/Sano s/p Glenn	Pre-Fontan	Yes
#13	2	F	T21 with a PDA	iNO testing	Yes
#14	4	M	TA + dTGA and a discrete CoA s/p Norwood/BTS s/p Glenn	Pre-Fontan	Yes
#15	5	M	HLHS, s/p Norwood/Sanos/p Glenn	Pre-Fontan	Yes
#16	5	M	HRHS + pulm atresia s/p BTS s/p Glenn s/p extracardiac fenestrated Fontan + LPA stent	Fenestrated Fontan	Yes
#17	5	M	T21 s/p PDA closure with an ASD and concern for pulmonary HTN	iNO testing	Yes
#18	7	M	TA s/p BTS + PDA ligation, s/p Glenn s/p extracardiac fenestrated Fontan	Fenestrated Fontan	Yes
#19	16	M	T21 + ASD, VSD + PDA with concern for pulmonary HTN	iNO testing	Yes
#20	4	M	DILV (S,L,L), s/p pulsutile Glenn with a PAB	Pre-Fontan	Yes
#21	16	M	DILV s/p extracardiac nonfenestrated Fontan with a Fontan stent and concern for PLE	Non-Fenestrated Fontan	Yes
#22	8	F	TOF with pulmonary atresia s/p RV-PA conduit s/p RV-PA conduit stent	RV volumes	Yes
#23	5	M	Heterotaxy, common AVC, s/p Glenn	Pre-Fontan	Yes
#24	13	F	Severe Discrete CoA	Coarctation	Yes
#25	11	M	HLHS s/p Glenn s/p fenestration Fontan	Fenestrated Fontan	Yes
#26	3	M	Dextrocardia, HLHS variant s/p Glenn	Pre-Fontan	Yes
#27	11	M	Severe Discrete CoA	Coarctation	No
#28	10	M	Severe Discrete CoA	Coarctation	Yes
#29	6	F	d-TGA s/p switch with concern for branch PA stenosis	PA stenosis	Yes
#30	9	M	HLHS s/p Norwood/Sano s/p Glenn	Fenestrated Fontan	No
#31	4	F	Pulmonary atresia with an IVS s/p ductal shunt s/p Glenn	Pre-Fontan	Yes
#32	33	F	Pulmonary atresia with an IVS s/p BTS s/p Glenn s/p Lateral Tunnel Fontan	Non-fenestrated Fontan	Yes
#33	7	F	s/p OHT	RVEDP, myocardial biopsy	Yes
#34	12	M	HLHS variant s/p PAB s/p Glenn s/p Lateral Tunnel Fontan	Fenestrated Fontan	Yes
