# Supplementary material for: Invasive cardiovascular magnetic resonance (iCMR) for diagnostic right and left heart catheterization using an MR-conditional guidewire and passive visualization in congenital heart disease
Source: J Cardiovasc Magn Reson. 2020 Mar 26;22:20. doi: 10.1186/s12968-020-0605-9 (PMC7098096; doi:10.1186/s12968-020-0605-9)
Supplement: Supplementary file 6 — Additional file 6. Supplemental information. [file 12968_2020_605_MOESM6_ESM.docx]

***Supplemental information***

**Abbreviations:**

Congenital Heart Disease (CHD)

Adult Congenital Heart Disease (ACHD)

Magnetic Resonance (MR)

Magnetic Resonance Imaging (MRI)

Invasive Cardiac Magnetic Resonance (iCMR)

Tesla (T)

Right Heart Catheterization (RHC)

Left Heart Catheterization (LHC)

Food and Drug Administration (FDA)

Conformité Européenne (CE)

Children’s Medical Center (CMC)

Balanced Steady-State Free Precession (bSSFP)

Flip Angle (FA)

Echo Time (TE)

Repetition Time (TR)Partial Saturation (pSAT)

IMproving Pediatric and Adult Congenital Treatment (IMPACT)

Single Ventricle (SV)

Bi-Ventricular (BiV)

Protein Losing Enteropathy (PLE)

Coarctation of the Aorta (CoA)

Inhaled Nitric Oxide (iNO)

Intra-Atrial Reentrant Tachycardia (IART)

Signal-to-Noise Ratio (SNR)

Specific Absorption Rate (SAR)

National Institutes of Health/National Heart, Lung, and Blood Institute (NIH/NHLBI)

Institutional Review Board (IRB)
Activated Clotting Time (ACT)

**Safety precautions**

***iCMR NIH training***

As a team, our CMR and interventional staff traveled to the National Institutes of Health/National Heart, Lung, and Blood Institute (NIH/NHLBI) in Bethesda, Maryland in April of 2016 to participate in the NIH-sponsored hands-on iCMR workshops to better understand the logistics of the procedure. We obtained Institutional Review Board (IRB) approval in early 2017. Prior to starting any procedures, safety protocols were written for each aspect of care. Furthermore, each member of staff involved in the procedure was identified as core-iCMR team and received specific safety training.

***Pre-iCMR team huddle***

Prior to each case, the CMR, catheterization, and anesthesia teams meet to discuss the case and strategies to improve safety, efficiency, and workflow. This short meeting helps reduce medical errors, highlights any areas of ambiguity, and is an opportunity to ensure consensus among team members.

***Anesthesia timeout and induction***

After obtaining consent in our perioperative area, the subject is transferred to Zone 3 (i.e. the MR control room in which only screened healthcare individuals and designated staff may enter) in the MR suite where the first timeout is performed to identify the subject and procedure by the attending anesthesiologist. At this time, anesthesia induction is initiated, adequate intravenous access is obtained, and the subject is intubated.

***Interventionalist timeout and access***

Once anesthesia induction is complete, the groin/neck are sterilely prepped and draped and the interventionalist performs a separate timeout. Percutaneous entry short sheaths were placed in the femoral vein (6-French), femoral artery (4-French), and internal jugular vein (6-French) when indicated using ultrasound guidance. At this time, an initial blood gas and activated clotting time (ACT) assessed to establish subject baseline and appropriateness to proceed. Heparin is administered for anticoagulation once access is complete.

***Zone 4 precautions***

Zone 4 represents the actual magnet room from which all ferromagnetic objects must be excluded. The catheterization team has prepared an “iCMR catheterization table bundle” for use in zone 4 containing no metallic or ferrous materials. Additionally, the magnet bore is prepared with sterile blue drapes and clear plastic lining to maintain sterility **(*Figure S1-C*)**. The vascular access equipment used in zone 3 includes ferrous needles, guidewires, forceps and scalpel. Prior to transfer to zone 4, an operating room style count is performed to ensure that no ferromagnetic equipment (needles/ wires/ scalpel/ forceps) is inadvertently transferred with the subject. Once in Zone 4, the femoral arterial line is balanced and connected to the Siemens Sensis (Version VD11, Erlangen, Germany) Hemodynamic recording system. Active MR imaging in Zone 4 is known to have noise implications for subjects and staff (interventional, anesthesiology and imaging teams). The subject uses standard noise protecting headphones with earplugs for protection. A standard in-bore subject to control room communication system is used if the procedure is performed without anesthesia. All monitoring is MR-conditional throughout this procedure.

***MR suite – Zone 3 to Zone 4 transfer***

Once access is established in Zone 3, the subject is transferred to the MR magnet in Zone 4. Initially, we were using an MR conditional medical trolley for transportation, however this required physically lifting the subject for transfer. We have since started utilizing the *Philips Ingenia MR table top* that directly connects to the MR magnet **(*Figure S1-A*)** which has increased our efficiency, decreased risk for contaminating sterility, and improved safety. Physical table top transfer of the subject is avoided with this table.

***Transfer from Zone 4 to x-ray fluoroscopy lab***

If intervention is indicated based on iCMR data, the subject is transferred to the adjacent x-ray fluoroscopy lab. Using the Philips MR table top, the subject is transferred from Zone 4, through two sets of double doors to the Phillips AlluraClarity catheterization laboratory (Philips Healthcare, Best, NL) located directly across from our Philips Ingenia 1.5 Tesla Magnet. The subject is then physically transferred to the x-ray fluoroscopy bed, and an additional timeout is performed by the interventionalist.

***Intra-procedural communication***

To improve safety measures and communication within the magnet, a multi-channel noise cancelling, wireless optical CMR communication system powered by Optoacoustic (***Figure S1-B***) was utilized. An infrared transmitter was installed within Zone 4 to allow for direct communication between the interventionalist, technicians, CMR team, and anesthesiologist while in MR interactive mode.

***Post-iCMR protocol***

If intervention is indicated based on flow, function, and pressure data, the subject is then moved to the x-ray fluoroscopy lab using a transfer table with the option for VesselNavigator^™^ system (Philips Healthcare) 3D overlay to help guide catheterization using the CMR angiographic images. This system allows for registration of these MR images using rotational x-ray fluoroscopy or, more simply, two AP fluoroscopy images greater than 30º apart. Previously placed fiducial markers are sometimes used to aid registration of images. After the intervention under x-ray fluoroscopy, the access sheaths are removed and hemostasis is achieved. The subject is extubated with standard post-cardiac catheterization procedure protocols followed.

If no intervention is needed, the subject is moved from Zone 4 to Zone 3, where access sheaths are removed, the subject is extubated, and transferred to the recovery unit. The subject is then observed for complications in an advanced nursing recovery unit for at least two hours. The subject is again assessed for complications via telephone encounter or clinic visit approximately three to seven days after discharge.

**Limitations**

***Visualization***

Our initial experience has shown that the procedure heavily relies upon proper simultaneous visualization of the balloon-tipped catheter, filled with dilute gadolinium contrast, and the subject’s anatomy. From our initial experience with these MR images, we see that the most ideal angle for proper simultaneously visualizing of the tip of the catheter, MR-conditional guidewire, and the subject’s anatomy is a TE 1.3ms, TR 2.7ms, pSAT angle 40^º^, and a FA of 35^º^. As only the tip of the guidewire and the tip of the balloon are visualized, the operator must always take care not to inadvertently loop either during catheterization. In order to achieve this, the operator must have strict knowledge at all times of the length of catheter advanced and the length of wire advanced from the catheter tip. In addition, balloon dilatation is only performed under visualization with real-time imaging as the tactile feedback of inflation with saline differs to inflation with carbon dioxide. Finally, as the real-time imaging involves multiple 2-dimensional planes and not projection imaging (as per standard fluoroscopic x-ray imaging), the possibility of the catheter/wire tip going out of plane exists. Hence catheter/wire manipulation in this circumstance will need to be ‘staccato’ as manipulation must be halted until the imaging plane is adjusted to allow catheter/wire tip visualization at all times.

***Communication from Zone 3 to Zone 4***

Communication between the MR technician (Zone 3) and interventionalist (Zone 4) within the magnet is an ongoing work in progress. The Optoacoustic headset system was acquired mid-way through this case series to aid with communication during the iCMR procedure. This technology has improved efficiency and decreased overall anesthesia time. However, we continue to struggle with intermittent feedback from the magnet into the headsets. From discussion with our catheterization team, this has been the largest barrier to efficiency.

***Spatial/temporal MR limitations***

Given the distinct but large artifact from the MR-conditional guidewire, we feel like this could be a major limitation for an operator to feel confident when addressing smaller structures such as AP and/or veno-venous collaterals and coronaries. In addition, the maximum temporal image acquisition time achieved is less then 6 frames per second (***Figure 2***). Unlike the x-ray fluoroscopy lab, which allows for consistent imaging during interventions, MR interactive mode’s image acquisition time may lead to delays in assessing or reacting to device deployment. On the other hand, MR’s improved soft tissue imaging quality may allow for better clinical outcomes and identification of complications not easily assessed with x-ray fluoroscopy. Overall, we felt that the spatial resolution was a bigger limitation to confidently entering smaller cardiac structures.

***MR unsafe catheterization equipment***

Most of the equipment used in a standard x-ray fluoroscopy lab is currently deemed MR-unsafe (e.g. braided catheters and standard guidewires). Currently, we primarily use iCMR for diagnostic and planning purposes. In addition to the development of further MR-conditional devices and equipment, smaller MR-conditional guidewire diameters are needed to help cross areas of severe stenosis. This need was most evident in *subject #27’s* case outlined in the results section. Additional and smaller size nanoparticle markers along the length of the MR-conditional guidewire may be required for improved conspicuity within the iCMR suite. Doing so will aid the interventionalist to readily identify any potential catheter and/or wire bends or loops within the blood vessels and heart chambers.

***Implanted device (steel coil) artifact***

The abundance of artifact from stainless-steel coils can disrupt image quality and limits MR based flow calculations, anatomical visualization and catheter motion during the diagnostic procedure. This was evident in *subject #30* as noted in the results section. This is a limitation to the procedure and may be a relative contraindication when screening subjects. We did not find a significant effect when scanning other subjects who had other MR conditional coils placed. However, current efforts within the institution are being made to reduce coil and metal related artifacts by introduction of newer sequences and patches to fix them.

***Multimodality table transport system***

Our current set-up involves the use of the Philips MR table top to obtain access prior to transferring into the MR magnet. This works very well when transferring within the MR suite, from Zone 3 to Zone 4, as the table directly connects to the MR magnet. The area for improvement and a limitation to our workflow occurs when transferring from the MR suite to the x-ray fluoroscopy lab. The current Philips MR table top used is not compatible with our catheterization lab and requires staff to physically transfer the subject to the x-ray fluoroscopy table top. Transfer of subjects between the CMR system and x-ray fluoroscopy lab adds the risks for dislodgement of devices attached to the subject, especially endotracheal tubes and vessel access sheaths. A potential improvement would be to incorporate a multimodality table transport system to our workflow to improve efficiency and safety for all iCMR subjects.

***MR-conditional guidewire under x-ray fluoroscopy***

As described in **Figure S4**, the MR-conditional guidewire has very limited visualization under x-ray fluoroscopy. Given the potential need for transfer to the x-ray fluoroscopy lab from the MR suite, we would consider this a possible limitation. If the wire is secured in a critical position, it is currently difficult to confidently confirm placement under x-ray fluoroscopy as the three passive MR markers at the tip of the wire are scarcely visible by low dose x-ray. To help lessen this limitation, one potential development would be to make the MR-conditional guidewire more lucent for use both in the CMR and x-ray fluoroscopy suites. Ideally, the entire wire shaft would be more radio-opaque and designed to allow for better tracking under x-ray fluoroscopy.
